# Some home-based self-managed rehabilitation interventions can improve arm activity after stroke: A systematic review and narrative synthesis

**DOI:** 10.3389/fneur.2023.1035256

**Published:** 2023-02-02

**Authors:** Kelly Westlake, Ruth Akinlosotu, Jean Udo, Andrea Goldstein Shipper, Sandy McCombe Waller, Jill Whitall

**Affiliations:** ^1^Department of Physical Therapy and Rehabilitation Science, University of Maryland, Baltimore, MD, United States; ^2^Health Sciences and Human Services Library, University of Maryland, Baltimore, MD, United States; ^3^Division of Health, Business, Technology, and Science, Frederick Community College, Frederick, MD, United States

**Keywords:** stroke, rehabilitation, home-based, self-managed, systematic review

## Abstract

**Background:**

There is an increased need for home-based, self-managed, and low maintenance stroke rehabilitation as well as interest in targeting the arm, which often lags behind leg recovery. Previous reviews have not controlled for concurrent standard of care and the ratio of self-managed care to therapist input.

**Objectives:**

To determine the effectiveness of home-based, self-managed and low maintenance programs for upper-limb motor recovery in individuals after stroke. A secondary objective explored the adherence to home-based self-managed programs.

**Data sources:**

We searched PubMed (1809-present), Embase (embase.com, 1974-present), Cochrane CENTRAL Register of Controlled Trials (Wiley), CINAHL (EBSCOhost, 1937-present), Physiotherapy Evidence Database (pedro.org.au), OTseeker (otseeker.com), and REHABDATA (National Rehabilitation Information Center). All searches were completed on June 9, 2022. Bibliographic references of included articles also were searched.

**Eligibility criteria:**

Randomized controlled trials (RCT) in adults after stroke, where both intervention and control were home-based, at least 75% self-managed and did not involve concurrent therapy as a confounding factor. Primary outcome was performance in functional motor activities after training. Secondary outcome was sensorimotor impairment. All outcomes after a retention period were also considered secondary outcomes.

**Data collection and analysis:**

Two review authors independently screened titles/abstracts, three review authors screened full papers and extracted data, and two review authors undertook assessment of risk of bias (i.e., allocation bias, measurement bias, confounding factors) using the NHLBI Study Quality Assessment Tool.

**Main results:**

We identified seven heterogenous studies, including five with fair to good quality. All studies had an alternative treatment, dose-equivalent control. Only one trial reported a positive, sustained, between-group effect on activity for the experimental group. The remaining studies reported seven interventions having a within-group training effect with three interventions having sustained effects at follow up. One study reported a between group effect on an impairment measure with no follow-up. Overall adherence rates were high, but three studies reported differential group rates. Compliance with daily logs was higher when the logs were collected on a weekly basis.

**Limitations:**

By excluding studies that allowed concurrent therapy, we likely minimized the number of studies that included participants in the early sub-acute post-stroke stage. By focusing on RCTs, we are unable to comment on other potentially promising home-based, self-managed single cohort programs. By including only published and English language studies, we may have included publication bias.

**Conclusions and implications:**

There is some evidence that a variety of home-based, self-managed training program can be beneficial after stroke. Future research could compare such programs with natural history controls. Clinicians might utilize home exercise programs with explicit directions and some form of weekly contact to aid compliance.

## Introduction

Stroke is the leading cause of disability worldwide (1). Of the annual incidence of stroke (~750,000) in the USA (2), about 60% fail to recover arm and hand use, resulting in reduced quality of life for survivors and caregivers (3). Improved survival rates, increased prevalence of risk factors, better long-term management, and an aging population are all predicted to contribute to an increasing prevalence of survivors (1). Thus, questions remain as to how we can best facilitate the rehabilitation of individuals with arm movement deficits and increase their quality of life over a longer time period. Principles of motor learning (4) and neuroplasticity (5), including repetition, feedback, motivation, and progression that are important for producing recovery, and best practice guidelines recommend a prolonged period of rehabilitation (6). Yet, rehabilitation intensity is not usually maximized to follow these principles (7). Despite research efforts to demonstrate ways to increase intensity in clinical settings (8), economic and recent pandemic pressures have led to shortened in- and outpatient therapy time; and the intensity needed for optimal rehabilitation is unlikely to be realized within these settings. Therefore, the ability for patients to train at home and manage their own rehabilitation duration, intensity, and progression, *via* effective self-management strategies, is vital.

In the last 20 years, there has been a growth of development and research in home-based rehabilitation programs. Three quite different approaches have emerged. One focuses on the benefits of therapists delivering the treatment at home, sometimes called early supported discharge (9). A second, focuses on tele-rehabilitation, defined by Appleby et al. as “the use of telecommunication, by either direct video or audio, to deliver rehabilitative interventions” (10). The third focuses on “self-managed” home-based rehabilitation, which we simply define as the implementation of therapy in the home either with or without low maintenance technology and/or assistive devices that is manageable by a patient with minimal help. Here we focus on the latter approach.

Early systematic reviews of the home-based, self-management approach revealed limited trials with relatively few of high quality and mostly with inconclusive results (11, 12). However, a Cochrane review of 14 studies, whose participants self-managed their rehabilitation, reported significant improvements in quality of life and self-efficacy but not on motor activity per se (13). More recently, Wong et al. (14) identified 15 good quality studies in chronic stroke survivors and determined that home-based, self-administered practice was no more effective than no intervention (5 studies) and that “structured” practice was no more effective than “non-structured” practice (10 studies) (14). Structured practice was defined by Wong et al. [(14); p.2] as a “task-oriented programme involving the use of technology and/or assistive devices in providing motivation, instruction, or feedback to people after stroke.” By inference, non-structured practice is essentially home exercise programs designed by therapists with no specific technology. The lack of definitive results from these studies is disappointing but, from an experimental motor learning perspective, it may be explainable by considering the eligibility criteria of the previous systematic studies.

To our knowledge, none of the reviews conducted to date have controlled for concurrent standard/usual care therapy to increase the internal validity of the studies reviewed. In a randomized controlled trial, the goal is to attribute improvement only to the experimental training program and not to any additional therapy (confound) that the participant is undertaking. In addition, concurrent therapy would dilute the effect of the experimental program because it is likely to be present for both experimental and control groups. Another potential confound is the amount of self-managed vs. therapist-led rehabilitation that occurs in a home-based trial. Trial participants need training at the start of the study, but the amount of therapist instruction received *throughout* the study could dilute the self-management aspect of the program and, if present for both groups, would further dilute the benefits of the experimental group. Taken together, controlling for these two criteria will plausibly increase the likelihood of finding positive results and, therefore, enable recommendations for effective home-based, self-managed rehabilitation for the upper extremity.

The main objective of this systematic review was to determine the effectiveness of home-based, self-managed rehabilitation interventions in improving upper limb function in individuals with stroke. We reviewed only randomized control trials in which both interventions were home-based and self-managed or in which the control group received no intervention or usual care. Based on our reasoning above, we anticipate that controlling for concurrent care and amount of therapist instruction may increase the chance of finding a positive result, that is a between group effect. It may also increase having a “partial positive” result (within-group effect for one group) or even a “neutral but positive” result (that is a within group effect for both interventions). In addition, since time on task is an important part of motor learning, and since adherence is a known problem in home-based programs (15, 16), a secondary objective is to explore the adherence to study protocols and to assess whether adhering to the planned schedule was related to either study outcomes or the methodology used to collect the adherence data.

## Methods

### Literature search

A medical librarian (AGS) conducted structured searches of the following databases: PubMed (1809-present), Embase (embase.com, 1974-present), Cochrane CENTRAL Register of Controlled Trials (Wiley), CINAHL (EBSCOhost, 1937-present), Physiotherapy Evidence Database (pedro.org.au), OTseeker (otseeker.com), and REHABDATA (National Rehabilitation Information Center). All searches were completed on June 9, 2022. Only articles published in English were included. The combined searches yielded 6,148 unique references. Bibliographic references of included articles were also searched for additional relevant studies. Abstracts and conference proceedings were also excluded if contacting the authors did not result in seeing a published paper.

References retrieved at least one term from each of two concepts: stroke and home-based rehabilitation (including terms such as telerehabilitation and home intervention). Search terms were adjusted for each database, and search strategies incorporated both keywords and subject headings (Appendix 1). Covidence was used to remove duplicates and to screen results.

### Eligibility criteria

#### Types of studies

We included randomized controlled trials (RCTs) where participants had been randomly assigned to a treatment or a control group. Control groups could have been receiving an alternative home exercise program, no intervention or usual care. We also included pilot RCTs, proof of principle or feasibility studies as long as they addressed efficacy in a randomized controlled trial.

#### Types of interventions

We included only upper extremity home-based interventions where experimental groups undertook all or a large majority of their training in their home and not in a community setting or clinic. Since the objective was to assess the effect of a largely self-administered intervention, the role of the research personnel was restricted to setting up the intervention, prescribing the treatment plan with little face-to face or only online interaction for the purpose of providing ongoing instruction/feedback. To this end, studies were included only if the research personnel interaction occurred <25% of the study intervention time. This percentage is a compromise between 50% [used by Wong et al. (14)] which is not a reflection of a largely self-administered program, and 0% which is unrealistic. Finally, while we did not have a specific type of intervention in mind, we rejected any that appeared to have a high cost or maintenance of technology required with expectation of encroachment of home space other than a computer or small robotic/orthotic devices for the arm.

#### Types of participants

Participants were adults (over 18 years), with a clinical diagnosis of stroke caused by either an infarct or hemorrhage. There was no restriction on chronicity or severity. We excluded studies that also included participants with diagnoses other than a stroke.

#### Types of outcome measures

To answer the question of effectiveness, we used the International Classification of Functioning, Disability and Health (ICF) that provides a framework for the description of health and health-related status (17). Within the ICF, components of functioning and disability are classified as (1) body structures/functions and potential impairments; (2) activity and potential activity limitation; and (3) participation in typical life tasks and the potential restrictions an individual may experience. For the purposes of this review, we included published outcome measures falling into the categories of activity and body structures/functions. We focused primarily on activity measures since these outcomes are likely to be most meaningful to stroke survivors. Body structures/functions were of secondary interest. We have not included outcome measures at the participation level, such as the Stroke Impact Scale, since improving these measures is likely to be attributable to other factors in addition to rehabilitation.

We defined the primary outcome measures for this study as those that measured functional arm motor tasks. This included, but was not limited to, objective measures such as Action Research Arm Test (ARAT); Box and Blocks Test (BBT), Jebsen-Taylor Hand Function Test (JTT), Nine Hole Peg Test (NHPT), Arm Motor Ability Test (AMAT), and Wolf Motor Function Test (WMFT-Timing; WMFT-Functional Ability). We also included questionnaires that focused on functional arm tasks and included Abilhand (ABIL) and Motor Activity Log (MAL-Quality of Movement or QM; MAL-Amount of Use or AU). We excluded any studies that had not included at least one of our selected primary outcomes. For all outcomes, we were primarily interested in measures taken immediately after the intervention. We also noted follow-up analyses where present because these indicate a greater probability of lasting improvements.

Secondary outcome measures represented impairment levels and included Active Range of Motion (AROM), Fugl-Meyer Assessment of Upper Extremity (FMA-UE), Grip strength (Grip) and Strength of specific joints.

To answer the secondary question of intervention adherence, we focused on compliance measures. These are typically reported by patients *via* a logbook or may be captured by a form of technology. We focused on the time-on-task reported as a percentage of the time planned. We did not consider the number of dropouts as a form of adherence because the reasons for dropping out may not reflect the attitude of the participant toward the intervention program. We compared the rates of adherence with outcome results to verify if there was a relationship; and with methodology used to ascertain whether this affected compliance.

#### Study selection

Two review authors (JU and JW) independently screened titles and abstracts of identified publications and eliminated irrelevant studies according to the pre-defined eligibility criteria (Box 1). Full texts for the remaining articles were obtained for further review and comparison with eligibility criteria (JU and JW). Conflicts between reviewers were resolved through discussion and, if required, through consultation with a third reviewer (KW).

Box 1 **Eligibility criteria for inclusion in review.**

**Inclusion criteria**

✓ Randomized controlled trial (not a systematic review) and could be a pilot or feasibility study.
✓ Adult stroke survivors only (no mixed populations).
✓ Upper extremity intervention only.
✓ Intervention and Control must be at least 75% self-managed (with some set up time).
✓ Self-managed Intervention/Control takes place in a home-based setting.
✓ Outcomes included activity measures of the upper extremity (e.g., WMFT; ARAT, MAL) as primary and could, but need not, include impairment measures of the upper extremity (e.g., FMA-UE) as secondary.

**Exclusion criteria**

✓ Robotic interventions that appear to consist of high cost/high maintenance technology since these are not feasibly placed in a home owing to cost and self-management limitations.
✓ Interventions designed specifically to be caregiver led because we are interested in the aspect of self-management with little need of help.

### Data extraction

Two review authors (from JU, RA, and JW), working independently, extracted data from the eligible studies using the Template for Intervention Description and Replication (TIDieR) checklist and guide (18). Categories were entered into Covidence and included the name of the intervention, rationale, materials, procedures, provider, mode of delivery, location, dosage, individualization, modifications, and planned and actual intervention adherence/fideility. In addition to the 12 items on the TIDierR checklist, information on sample size, study participants, outcomes measured (including time points), and intervention efficacy were included. Efficacy was defined as improvements on activity or body structure/functions clinical outcomes related to upper extremity function. Discrepancies in data extraction were resolved *via* discussion with involvement of the third review author, as necessary. Prior to commencing data extraction, we piloted the adapted TIDieR checklist to ensure that the review authors were using the tool comparably.

### Risk of bias

Two review authors (RA, JW) independently assessed the methological quality of each article that met eligibility criteria. The method used was the National Health Lung Heart and Blood Institute (NHLHBI) Quality Assessment Tool for Randomized Controlled Trials (19) that assesses (1) allocation bias, (2) measurement bias and (3) confounding factors that reduce the internal validity of the outcomes based on 14 questions. We chose this tool because it included questions we thought were important such as adherence and concurrent interventions and it could be entered into Covidence. Decisions for each question were Yes/No/Other with other including Cannot Determine (CD), Not reported (NR), and Not Applicable (NA). Any disagreements were resolved by discussion between the two reviewers and a third reviewer (KW). Overall ratings of good, fair and poor quality were derived, independently, by the same review authors (RA, JW) using the guidelines of the NHLHBI tool that include a consideration of internal validity and fatal flaws in addition to summary score (19). The questions are not intended to create a list that is simply tallied up to arrive at a summary judgement of quality but rather to assess the risk of bias with high risk translating to poor quality and low risk translating to good quality. Fatal flaws such as high dropout rates, high differential rates or no Intention to treat analysis indicate that the risk of bias is significant. Disagreements in overall ratings were discussed and resolved with the addition of a third reviewer (KW).

### Planned synthesis methods

For effectiveness, we planned to conduct separate meta-analyses on our primary and secondary outcomes by combining activity level outcomes for the primary outcome and using the FMA-UE for the secondary outcome. For adherence, we planned to correlate the rate with the highest effect size of our primary outcome in each study. We also planned to compare the methodology used with the adherence rate and compliance.

## Results

### Search results

**Figure 1:**
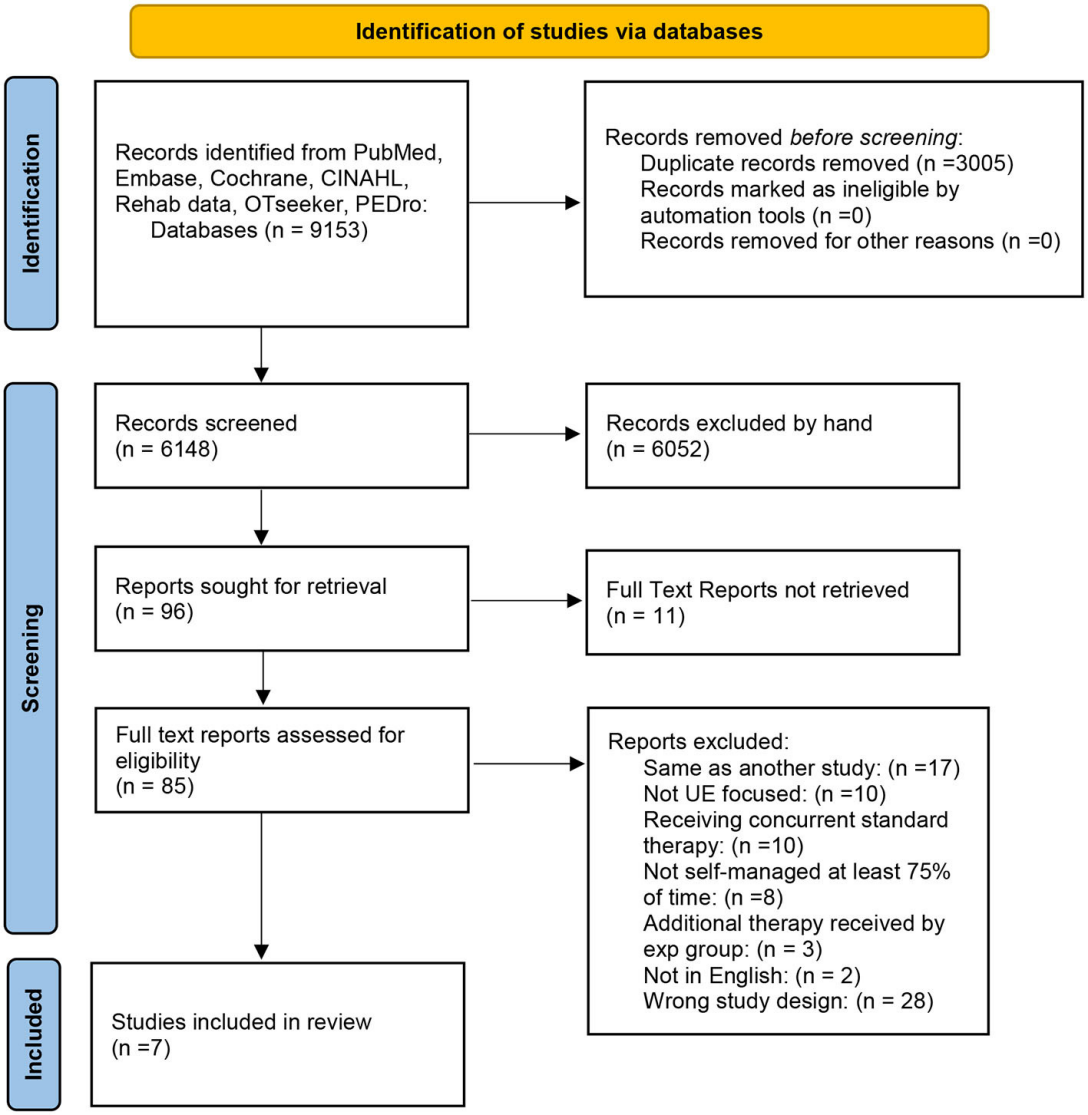
Prisma flow chart.

As outlined in the PRISMA flow chart (Figure 1), our initial search procedure yielded 9,057 studies. After removing duplicates, 6,148 studies were screened for title and abstract. Subsequently 85 full-text studies were eligible for full review. Full text review resulted initially in a total of eight articles. Manual searching of these included studies did not identify any additional eligible studies that had not been reviewed. Because one of our eligibility criteria stated that no physical therapy or intervention should be in progress concurrent to the home-based intervention, we contacted the authors of four of the studies that had not explicitly stated that no concurrent physical therapy was being experienced. Based on this information, we excluded one further study in which 38% of the participants were undergoing concurrent therapy with, importantly, twice as many in one group vs. the other (20). Another study had 12% of the participants undergoing concurrent therapy, but this number was split evenly between the two groups so we retained that study (21). Thus, the final included number of studies was seven.

### Participants

A summary of the population and study design characteristics for each included study is provided in Table 1. Across studies, the groups of participants had a mean age of 58.8 years (range 52.2–64.9) and a mean time post stroke of 3.17 years (range 0.32–7.7). None of the studies reported within study group differences at baseline except for sex. The mean percentage of females across all groups and studies was 58% (range 22–88). Two studies had different ratios of female to male between study groups (23, 24). For severity of impairment, categorization was based on the Fugl-Meyer Upper Extremity Scale (28, 29) or the Modified Rankin Scale (30). Five studies targeted mild, mild-moderate and moderately impaired participants (21, 23–25, 27), one study targeted moderate to severely impaired participants (26) and one study reported participants from all classifications (22).

**Table 1:** Characteristics of randomized controlled trials of included studies.

**References, country**	**Sample n E vs. C**	**Age years E vs C (SD)**	**Sex F E vs. C (%)**	**Time past stroke years E vs. C (SD)**	**Severity E vs. C (SD/R) MRS or FMA**	**Total hours day-min X week #weeks**	**Intervention**	**Control**	**Outcomes Bold = Primary Assessments F/U weeks post randomization**	**Main results**
dos Santos-Fontes et al. (22) Brazil 100% self	10 vs. 10	52.2 (11.1) Vs. 59.1 (11.1)	5(50) Vs. 6(60)	3.8 (4.5) vs. 3.3 (2.1)	MRS (49–64) vs. (48–64) Mild to Sev.	70 h 150 min/d 7d/wk 4 wks	Active repetitive peripheral nerve somatosensory stimulation (wrist-band) + JTT tasks	Sham stimulation using wristband + JTT tasks	**JTT** **Post** F/U 16 wks.	Positive: JTT improved for experimental over control; retained at F/U
Michielsen et al. (23) Netherland 85% self	20 vs. 20	55.3 (12.0) vs. 58.7(13.5)	13(65) vs. 7(35)	4.7(3.6) vs. 4.5 (2.6)	FMA UE 39.7 (14.1) vs. 36.4 (14.7) Mod	30 + 6^*^ h 60 min/d 5 d/wk 6 wks	Mirror training- observing stronger arm + instructional booklet	Training observing both arms + instructional booklet	**FMA UE**, Grip force ARAT, ABIL, Stroke- ULAM **Post** F/U 24 wks.	Partial: FMA-UE improved for experimental only, lost at F/U. Neutral negative: all other variables.
Nijenhuis et al. (24) Netherland 100% self	9 vs. 10	58 vs. 62	2(22) Vs. 7 (70)	0.92 vs. 1	FMA UE 59.1 (48–65) vs. 62 (54–70) Mild	18 h 30 min/d 6 d/wk 6 wks	Computer games w. passive dynamic wrist/hand orthosis & Saebomas.	Prescribed conventional exercises in an exercise book	**ARAT** FMA UE, MAL, Grip **Post** F/U 15 weeks	Partial: ARAT and FMA UE improved for control only retained at F/U for ARAT only. Neutral negative: all other variables
Rand et al. (25) Israel 100% self	13 vs. 11	59.1(10.5) vs. 64.9(6.9)	4(31) Vs. 5(46)	1.63 (0.92) vs. 1.08 (0.5)	FMA UE 35.4 (20–55) vs. 41.3 (21–55) Moderate	30 h 60 min/d 6 d/wk 5 wks	Videogames (Sony PlayStation 2 EyeToy video-capture). For some Microsoft Xbox Kinect too.	Traditional exercises and activities from the validated Graded Arm Supplementary Program (GRASP).	**ARAT**, MAL-AU, BBT, **Post** F/U 9 wks.	Neutral: **A**RAT improved at post and F/U. Neutral: MAL improved at F/U only. Neutral Negative: BBT
Sullivan et al. (26) USA 100% self	20 vs. 18	61.6 vs. 59.5	7(35) Vs. 4(22)	7.7 vs. 6.6	FMA UE 29.1 vs. 27.4 Moderate/Severe	20 h 60 min/d 5 d/wk 4 wks	Sensory amplitude electrical stimulation via a Glove stimulator during task-specific arm exercises (from COPM)	Sham glove with task-specific arm exercises (fromCOPM)	**FMA UE**, **AMAT**, MAL-14, **Post**	Partial: AMAT improved for experimental only. Neutral Negative: all other variables.
Wolf et al. (27) USA 89% self	51 vs. 48	59.1 vs. 54.7	26 (51) vs. 17(35)	0.32 vs. 0.35	FMA UE 34.1 (12.1) vs. 33.3 (12.0) Moderate	120+2^**^ h 180 min/d 5 d/wk 8 wks	Robot arm/computer (hand mentor pro) + Home Exercise program	Home exercise program alone	**ARAT**, WMFT-T; WMFT-FA, FMA UE **Post**	Neutral: ARAT, WMFT-T; FA and FMA UE improved at post.
Zondervan et al. (20) USA 100% self	9 vs. 8	60 vs. 59	5 (56) vs. 5 (63)	5.3 vs. 3.2	FMA UE 53.8 (8.9) vs. 56.4 (6.3) Mild	9 h 60 min/d 3 d/wk 3 wks	MusicGlove instrumented for finger movement therapy in time with music.	Conventional tabletop exercises	**BBT**, MAL QM & AU, NHPT, ARAT **Post-F/U** 7 wks.	Partial: MAL AU and QM improved at post/F/U assessment. Neutral negative: all other variables.

ARAT, Action Research Arm Test; BBT, Box and Blocks Test; COPM, Canadian Occupational Performance Measure; FMA UE, Fugl Meyer Assessment Upper Extremity; JJT, Jebsen–Taylor Test; MAL-14 Motor Activity Log 14 questions; MAL AU, Motor Activity Log Amount of Use; MAL QM, Motor Activity Log Quality of Movement; MRS, Modified Rankin Scale; NHPT, Nine Hole Peg Test; SIS, Stroke Impact Scale (16 questions); WMFT-T, Wolf Motor Function Test-T, Timing; WMFT-FA, Wolf Motor Function Test-Function.

Positive, Between group difference; Neutral, Both groups improve, no difference; Negative, neither group improves, no difference; Partial, One group has within group improvement.

* Six hours of training (one per week) were given by therapists in a center.

** Two hours of training (.25 h per week) were given by therapists in a center.

### Study designs

The studies included three statistically powered randomized trials (23, 26, 27), three pilot randomized trials (21, 24, 25), and one proof of principle randomized trial (22). Four studies reported immediate post training and follow-up assessments (22–25). Two studies reported immediate post training assessments but no follow-up (26, 27). One study also reported one post-training assessment, but it was a month after the end of training (21), thus providing a more sustained effect than an immediate post training –test.

The experimental interventions were of three general types: (1) neuromotor stimulation delivered along with home arm exercises either concurrently (26) or sequentially (22); (2) computerized game training with small orthotic (24) or robotic devices (21, 27), the latter combined with a home exercise program, or video-games (25); and (3) mirror training (23). See Table 1 for more details. Control group programs were of alternative types that were either (1) the same as the experimental group with one difference, for example sham stimulation with similar conventional exercises for each group (22, 26), bilateral visual cueing (vs. the experimental unilateral) mirror training with similar conventional exercises for each group (23), the same home exercise program as the experimental group but for a longer duration (27); or (2) different from the experimental group for example prescribed home exercise programs (21, 24) or a specific prescribed program, called Graded Repetitive Arm Supplementary program (31) also known as GRASP (25).

Selection of outcome variables was also not consistent across the studies. The primary variables in five studies were activity measure (21, 22, 24, 25, 27), while one study evaluated impairment or body/structure/function levels (31), and one study focused on both activity and impairment outcomes (26). For the activity variables, the focus of this review, five studies included the ARAT, four included the MAL, two included BBT and one each included JTT, AMAT, NHPT and ABILHAND. For our secondary measure of impairment, five included the FMA-UE and two also included grip strength.

### Risk of bias

The methodological quality of the included studies is summarized in Table 2. Of the seven studies, four demonstrated high methodological quality (22, 23, 25, 27), one was of moderate-high quality (26), and two were of lower quality (21, 24). Major problems of those ranked as having poor quality were lack of blinded assessors (24) and inadequate randomization (21). In addition, some studies had problems with a different level of adherence between the two groups (22, 24, 25), and a differential amount of missing data between groups from participants who did not report in study logs (21). Adherence is discussed in detail later as it is our secondary research question.

**Table 2:** NHLBI study quality assessment tool categorization of included studies and level of evidence.

**References**	**NHLBI assessment questions**														**Total yes**	**Total no**	**Other**	**Rate**
	**Q1**	**Q2**	**Q3**	**Q4**	**Q5**	**Q6**	**Q7**	**Q8**	**Q9**	**Q10**	**Q11**	**Q12**	**Q13**	**Q14**				
dos Santos-Fontes et al. (22)	Yes	Yes	Yes	Yes	Yes	Yes	Yes	Yes	No	Yes	Yes	No	CD	Yes	11	2	CD	Good
Michielsen et al. (23)	Yes	Yes	Yes	No	Yes	Yes	Yes	Yes	Yes	Yes	Yes	Yes	CD	Yes	12	1	CD	Good
Nijenhuis et al. (24)	Yes	CD	Yes	No	No	Yes	Yes	Yes	No	Yes	Yes	No	CD	NR	7	4	CD; CD; NR	Poor
Rand et al. (25)	Yes	Yes	Yes	No	Yes	Yes	Yes	Yes	No	Yes	Yes	No	Yes	Yes	11	3		Good
Sullivan et al. (26)	Yes	No	CD	CD	Yes	Yes	Yes	Yes	No	Yes	Yes	Yes	CD	Yes	9	2	CD; CD CD	Fair
Wolf et al. (27)	Yes	Yes	Yes	No	Yes	Yes	Yes	Yes	Yes	Yes	Yes	Yes	Yes	Yes	13	1		Good
Zondervan et al. (20)	Yes	No	No	No	Yes	Yes	Yes	Yes	No	Yes	Yes	No	No	NR	7	6	NR;	Poor

Assessment Questions: 1. Was the study described as randomized? 2. Was the method of randomization adequate? 3. Was the treatment allocation concealed? 4. Were study participants and providers blinded to treatment group assignment? 5. Were the people assessing the outcomes blinded to the participants' group assignments? 6. Were the groups similar at baseline on important characteristics that could affect outcomes? 7. Was the overall drop-out rate from the study at endpoint 20% or lower of the number allocated to treatment? 8. Was the differential drop-out rate at endpoint 15% or lower? 9. Was there high adherence to the intervention protocols? 10. Were other interventions avoided or similar in the groups? 11. Were outcomes assessed using valid and reliable measures? 12. Did the authors report that the sample size was sufficiently large? 13. Were outcomes reported or subgroups analyzed prespecified? 14. Were all randomized participants analyzed in the group to which they were originally assigned?

Other: NR, not reported; CD, cannot determine.

### Effectiveness

Our primary question was to determine the effectiveness of home-based, self-administered interventions based on improvement of an activity outcome. The small sample size, coupled with the clinical heterogeneity of the studies including design, interventions, comparators, patient severity, chronicity and risk of bias precluded conducting meta-analyses (32). Therefore, we present a narrative synthesis of the seven studies. The main results of each intervention are summarized briefly in Table 1 but expanded upon in Table 3 where the results of the experimental intervention and the control intervention are presented independently.

**Table 3:** Results for each home intervention program considered independently.

**References**	**Intervention**	**Activity variable**	**Prime endpoint results**	**F/U after PE**	**Second variables**	**Prime endpoint results**	**F/U after PE**
Dos Santos-Fontes et al. (22), Exp	RPSS + Motor training	JTT	>	>	None		
Dos Santos-Fontes et al. (22), Con	Sham SS + Motor training	JTT	<	<	None		
Michielsen et al. (23), Exp	Mirror train train watching non-paretic arm	ARAT ABIL	- -	- -	FMA GRIP	> -	- -
Michielsen et al. (23), Exp	Train watching both arms	ARAT ABIL	- -	- -	FMA GRIP	< -	- -
Nijenhuis et al. (24), Exp	Computer + Wrist and hand orthosis, saebomas and gaming exercises	ARAT B&B MAL-A MAL-Q	- - - -	-	FMA GRIP	- -	- -
Nijenhuis et al. (24), Con	Conventional home exercise	ARAT B&B MAL-A MAL-Q	+ - - -	+	FMA GRIP	+ -	- -
Rand et al. (25), Con	Video games	ARAT MAL-A MAL-Q	+ - -	+ +	None		
Rand et al. (25), Exp	GRASP	ARAT MAL-A MAL-Q	+ - -	+ +	None		
Sullivan et al. (26), Exp	Glove stim/COPM	AMAT MAL-14	+ -	None	FMA	-	None
Sullivan et al. (26), Con	Sham stim/COPM	AMAT MAL-14	- -	None	FMA	-	None
Wolf et al. (27), Exp	Computer/Robot + HEP	ARAT WMFT-T WMFT-FA	+ + +	None	FMA	+	None
Wolf et al. (27), Con	HEP alone	ARAT WMFT-T WMFT-FA	+ + +	None	FMA	+	None
Zondervan et al. (20), Exp	Music glove plus computer exercises	ARAT B&B NHPT MAL-A MAL-Q	- - - + +	None	None		
Zondervan et al. (20), Con	Tabletop exercises	ARAT B&B NHPT MAL-A MAL-Q	- - - - -	None	None		

ABIL, Abilhand; ARAT, Action Research Arm Test; BBT, Box and Blocks Test; COPMP, Canadian Occupational Performance Measure; FMA UE, Fugl Meyer Assessment Upper Extremity; JJT, Jebsen–Taylor Test; MAL-14, Motor Activity Log 14 questions; MAL-A, Motor Activity Log Amount of Use; MAL-Q, Motor Activity Log Quality of Movement; NHPT, Nine Hole Peg Test; WMFT-T, Wolf Motor Function Test-Timing; WMFT-FA, Wolf Motor Function Test-Function. >, between group effect over other study arm; +, within group effect; -, no effect; <, between group effect favors the other arm.

For activity-based arm measurements, studies are summarized below based on the following statistical results: between-group improvement; experimental within-group improvement; both experimental and control within-group improvements; no effect; and only control with-group improvement. Dos Santos et al. reported a between-group effect in favor of the experimental group on the JTT immediately post-training and at follow-up (22). Sullivan et al. reported a within-group effect for the experimental group only on the AMAT at the post-training assessment, but there was no effect on the MAL and no follow-up assessment (26). Zondervan et al. reported a within-group effect for the experimental group only on the MAL-AU and MAL-QM at 1 month after the end of training but no effects on the BBT, ARAT, NHPT and no further testing. Rand et al. reported within-group improvements for both groups on the ARAT at post-testing and follow up and on the MAL-AU at follow up only (25). Wolf et al. also reported within group effects for both groups on the ARAT, WMFT-T and WMFT-FA but no further testing (27). Michielsen et al. reported no effects on either group on the ARAT or ABIL at post-test or follow-up (23). Finally, Nijenhuis et al. reported a within group effect for the control group only on the ARAT at post-test and follow-up, but no effects on BBT, MAL-AU or MAL-QM (24). In summary, only one study (22) reported efficacy over the control intervention, two studies (21, 26) reported an improvement for the experimental group only, two studies (25, 27) reported improvements for both experimental and control interventions, one study (23) showed no improvement for either group, and one study (24) showed an improvement for the control group only.

Four studies included measurements of our secondary variables of arm impairment (23, 24, 26, 27). One study reported an experimental between-group advantage on the FMA-UE at post-intervention testing that was lost at follow-up and no improvements on the GRIP (23). One study reported within-group improvements on the FMA-UE for both intervention and control groups at post-testing (27). One study reported no improvement for either group on the FMA-UE (26). The final study reported a within group improvement for the control group on the FMA-UE (lost at follow-up) and no improvement on the GRIP (24).

### Adherence

Our secondary question was to explore the adherence to the home-based self-managed programs. Table 4 presents the adherence rate and methodology reported from each study. All relied on self-report except Sullivan et al. (26) who used a stimulator compliance meter for both groups. Two other studies (21, 24), had a device that measured only the experimental group although they relied on a daily log. Among all studies, there was an average adherence rate of (92.2%) with adherence of experimental vs. control groups varying little (92.3–92.1% respectively). Inspection of differences in adherence within a study provides a different perspective since at least 3 of the studies reported significantly different adherence rates between the groups with one favoring the experimental group (22) and two favoring the control group (24, 25). The remaining studies (21, 23, 26, 27) reported no significant difference in group adherence. However, Sullivan et al. reported a large difference favoring the experimental group and lower compliance in the control group versus experimental group despite non-significant findings (26). Zondervan et al. reported a marked difference in the number of participants in each group who provided adherence data thus questioning the validity of the adherence data in this study (21). Dos Santos et al. also commented on participants failing to fully complete their adherence reports (Table 4) (22). All but two studies (21, 22) noted that they collected their adherence data on a weekly basis.

**Table 4:** Adherence to self-managed training.

**References**	**Planned time (Hours)**	**Adherence rate (%) Exp**	**Adherence rate (%) Con**	**Sig. Dif**	**Data method**	**Recording compliance**
dos Santos-Fontes et al. (22)	RPSS = 56 MT = 28	98.2 80.0 = 89.1	82.9 40.7 = 61.8	*p* = 0.003 *p* = 0.002	Daily log for RPSS/MT reported after intervention. Weekly call	Patients reported that they often forgot to register motor training and use of RPSS. No other details.
Michielsen et al. (23)^*^	30	100	100	No value given	Daily diary inspected weekly in rehab center	No missing adherence data reported.
Nijenhuis et al. (24)	18	65.6	105	*p* = 0.025	Daily diary plus weekly team visits for all. EG: Recorded on device.	No missing adherence data reported
Rand et al. (25)	30	62.7	91.3	*p* = 0.014	Daily log transmitted weekly	No missing adherence data reported.
Sullivan et al. (26)^#^	28 40 sessions	106 42.4	80 31.9	*p* = 0.114 p = 0.396	Stimulator compliance meter weekly call	No missing adherence data reported CG biased to low
Wolf et al. (27)	120	111.8	116.3	*p* = 0.68	Daily diary reported weekly by phone or email.	No missing adherence data
Zondervan et al. (20)^∧^	9	111.1	90	No value given	Daily log reported after intervention Weekly call	Data from log not available from 2/9, 4/8 EG and CG respectively.
Study Average Range	Overall 92.2 75.5–114.1	92.3 62.7–111.8	92.1 61.8–116.3			

Where adherence exceeds 100%, the participant did more than planned. If these cases are capped at 100% the mean drops to 88.6%.

* Quantitative data not reported in the paper; only the average as 30 across groups. #Recorded in two ways; amount of time and sessions completed.

∧ No difference stated in paper, but no p value given. The compliance with reporting is biased across groups. EG, experimental group; CG, control group.

## Discussion

The aim of this systematic review was to determine the effectiveness of home-based, self-managed low tech/cost rehabilitation interventions on improving upper limb functional activity in individuals with stroke. Seven heterogenous studies met our criteria. All had dose-matched alternative home based, self-managed control programs. Only one study (22) demonstrated a significant result for the experimental over the control group. Within study changes showed that a further seven interventions produced a positive effect immediately after the training and three of these were maintained after a retention period. In addition, we explored whether these self-managed interventions had good compliance. Adherence was high across studies but at least three studies (22, 24, 25) had significantly less adherence for one group which could have affected the outcomes. Adherence was higher for those studies that collected data on a daily or weekly basis. Four of the studies (22, 23, 25, 27) were judged to have a low risk of bias but all studies had groups equal for sample size, age, chronicity, severity and baseline characteristics with two studies having unequal female/male ratios.

### Study selection

We anticipated that a larger number of studies would meet our eligibility criteria given that an earlier but recent study (14) with similar eligibility criteria had identified 15 studies. We attribute the paucity of studies largely to our eligibility criteria of (a) excluding studies where patients were allowed to continue with other motor therapy while participating in the study and (b) imposing a 75% minimum on the percent hours where patients were self-administering their treatment vs. trainer led. Many of the studies we evaluated in the full paper review were rejected on one or both criteria (Figure 1) and many more in the abstract review. We consider the inclusion of both criteria as a strength of this review because they affect the internal and external study validity, respectively as stated in the introduction.

### Effectiveness of home-based, self-managed programs

We anticipated that the inclusion of our restrictive eligibility criteria could result in more positive trials and/or meta-analyses. This did not occur. Even on an individual study basis, we identified only one study (Dos Santos) with an experimental intervention that performed better than a typical set of home-based exercises that could easily be made available to patients. The lack of positive results are plausibly a consequence of pre-study choices of interventions and the appropriate control groups.

Since the results of six experimental interventions were no better than the control interventions, it is possible that the structured part of the interventions may have been insufficient. In other words, the experimental interventions may not have been designed with enough or the right type of support to enable individuals to progress. Essential components of motor learning include amount and variation of practice, feedback, knowing when to progress and motivation to practice (4). One advantage of technology, is that feedback and progression suggestions (instruction) can be built into computer software to guide participants and motivate them remotely. This kind of structured experimental home-based practice, which applied to 5 of our 7 interventions (21, 24–27), was expected to be more effective than non-supervised home-based practice where the participants make all the decisions based on prior instructions and a home exercise program from a therapist. The fact that it was not suggests that other factors may be involved.

It is our contention that the choice of control group activities may have contributed to reducing the likelihood of demonstrating superiority in at least three of the six studies that did not show a positive result for activity measures. Descriptions of control groups with so-called conventional, standard or usual care are generally poorly defined (33). However in the context of these three studies (23, 26, 27), there was an effort to control for as many confounding factors as possible by including the same type of motor training in the control group as the experimental group. This is understandable for the stimulation study (26) since it was contrasting actual nerve with sham stimulation but this also occurred with the mirror training study (23) which differed only by whether the participant was looking at the non-paretic hand in a mirror versus both hands as normal; and the Home Mentor Pro (27) which substituted 1 h of computer/robot training with a home exercise program and had 2 h of home training per session in common. These studies may require a larger sample size for statistical power because only one factor (or segment) of the training is changed; that is, the control group is doing much the same motor practice as the experimental group along with having the same weekly contact support. In this situation, we might expect neutral results where both training methods cause a similar response whether it be neutral positive (within-group changes) or neutral negative (no group changes). Indeed, two of the three studies in this control subgroup reported neutral positive trials (23, 27) and one had mostly neutral results (26) which supports this idea.

With the same logic, the three studies that chose to compare dissimilar training programs might be more likely to show non-neutral results. In fact, two of these studies showed a positive within group effect with one for the control group (24) and the other for the experimental group (21) although the latter result occurred only for one of four activity-based outcomes. The third study showed neutral and positive within group effects indicating the effectiveness of both interventions (25).

Another plausible reason for the lack of definitive results could be that all study populations but one were in the chronic post-stroke stage in which participants were likely to have stabilized and no longer had the benefit of either spontaneous recovery or the more ideal recovery time for neuroplasticity to occur (34). However, there are certainly a large number of lab-based trials that do show effects in a chronic population (often of both groups) so this is less likely as an explanation (35).

A final reason for the lack of superiority of the experimental over control groups could be that the interventions were simply not run for a long enough time. A recent meta-analysis found that greater amounts of a specific intervention failed to show any advantage on arm activity measures *unless* there was a *large* time difference between the two groups (36). This suggests that time/amount of training is important but the authors were unable to provide a specific hourly recommendation. An early meta-analysis by Kwakkel et al. (37) suggested that 16 h of training is a minimum requirement to see an effect within the first 6 months after stroke. One might reasonably expect a longer time in chronic stage but in the studies reviewed here, only Zondervan et al. had <16 h planned and actually did see some benefit (in one out of 4 outcome variables) in the experimental group (21). Nijenhuis et al. planned for 18 h but the experimental group achieved only 11 h, which could explain why they did not improve even though the control group, which did improve, achieved 19 h (24). The three studies with positive or neutral positive results had a range of 30 to 120 planned hours (Table 3) suggesting that amount of hours could be a factor for improvement.

In summary, this review revealed little evidence that experimental interventions are better than alternative conventional programs. On the other hand, there was some evidence that eight different intervention programs (including the one from the positive trial) were able to significantly improve the participants activity performance after training and four of these effects were sustained. These two statements are strikingly similar to those made after a recent review of large multicenter stroke rehabilitation trials (35) indicating that the question of determining experimental interventions that are better than conventional/alternative interventions is not restricted to home-based, self-managed intervention RCT trials.

### The role of adherence

Attaining adherence to a planned training schedule is an important issue for home-based, self-managed interventions if repetition and time on task are important for learning (4). In this review, overall adherence was high at 92% across studies and with six out of 14 study arms exceeding their planned duration. Even capping those six arms at 100%, reduced the rate only to 89% with four studies at 90% or above (Table 3). Ensuring high adherence and/or similar adherence between the groups is advisable since it increases the internal validity of a study. Furthermore, adherence in these studies required the motivation of a weekly contact to all participants. For most studies (except Wolf et al.), there was a lack of clarity as to the amount of weekly contact that involved instruction (giving feedback and adjusting progression) vs. time devoted to motivating participants to fill in the study log, do their practice, and report adverse events.

When looking at the subgroup of studies with differential between group adherence, all below the median of 90%, there are some concerns about the groups with a lower adherence. For example, Rand et al. demonstrated within group effects for both videogaming and control GRASP protocols but the adherence rate for the GRASP protocol was significantly higher by 29%, which may have influenced results for the control group (25). Similarly, Nijenhuis found a positive effect for conventional home exercise compared to a computerized gaming system with a wrist orthosis (24). Since the control group had a significantly higher adherence rate of 39% greater than the experimental groups, differences in adherence may explain their finding and lowers the internal validity of the study. Only Dos Santos et al. demonstrated that their positive finding was not influenced by differential adherence rates (22). Two other studies (21, 26) also had a large but non-significant difference between groups (over 20% and in favor of the experimental group). In both cases there were issues with the adherence or compliance of the control groups that may have lowered performance of the control groups (Table 4).

As is well known, self-reported adherence data are subjective and prone to error unless there is a technical method involved to record the movement. Even then participants can forget to switch the meter on or switch it on and do nothing. In this review, only one study (26) had a compliance meter that both groups could use. We cannot comment on the veracity of the technical methods of recording but we do note that the two studies which reported problems with participants not completing logs, both collected the data at the end of the intervention and not on a weekly basis as the other studies did (21, 22). Thus, the weekly contact with the participants (common to all studies) may not have adequately motivated participants to record their time if they were not required to hand in the log weekly.

### Limitations

There are several study limitations that should be mentioned here. First, one consequence of the exclusion of studies with concurrent therapy is that the number of studies including participants in the early sub-acute post-stroke stage was likely minimized. Thus, the generalizability of our findings may have been reduced. Second, by focusing only on RCTs, there may have been additional promising home-based, self-managed single cohort programs that we missed in this review. Third, by not including un-published studies or non-English language, we included publication bias. Finally, the variability of the reported studies precluded a meaningful statistical analysis of the studies as a group.

### Future research recommendations

For researchers interested in demonstrating effective home-based, self-managed low cost/technology, this review has revealed one intervention RPSS + conventional motor training that appears to be beneficial and this needs to be followed up and tested on other outcome variables. The review also identified several other potentially effective programs. One recommendation, regardless of which type of program is designed, to promote self-managed learning, is to compare it to natural history controls rather than to traditional physical therapy home programs with equal support/motivation. This strategy will provide a more realistic and pragmatic trial with increased external validity (albeit with lower internal validity), and more likelihood of a positive trial. It will also be less expensive to conduct although use of a cross-over or delayed-entry design would be helpful for recruitment. Such a trial will be easier to conduct in the chronic stage. In the sub-acute stage, with concurrent standard therapy administered to all participants, substituting the new intervention for at least part of the standard care, and keeping the dose equal would be ideal if local health officials will allow this. To achieve high adherence in the experimental group we recommend considering the use of a weekly check-in including transmittal of the daily log as well as the use of a technological method if plausible.

### Clinical implications

The fact that this review, like others, has found little evidence that experimental interventions are better than traditional home-based exercise programs is good news for clinicians. We identified eight independent home-based, self-managed intervention programs that demonstrated a within or between group benefit after use. Importantly, three of these programs were control interventions consisting only of traditional/conventional home exercise programs. One is the well-validated GRASP program (31). Therefore, clinicians can feel comfortable with sending progressive activity and exercise programs home with patients along with good written instructions. In addition, to produce good adherence to these programs and to keep patients motivated and progressing it would be advisable to implement regular (i.e., weekly) contact with the possibility of some brief tele-rehabilitation. Volunteers and aides might be useful in this regard although a therapist would need to supervise.

## Conclusion

This review found little evidence that purposively designed home-based, self-managed arm rehabilitation is any better than a conventional home exercise program that has the same dose and motivational support. However, eight interventions did show potential for improving motor ability including two nerve stimulation studies with home exercises, three home exercise programs, video games, a music glove, and a robotic arm with home exercises prior to home exercise programming.

## Data availability statement

The original contributions presented in the study are included in the article/Supplementary material, further inquiries can be directed to the corresponding author.

## Author contributions

KW, SW, and JW contributed to the conception, design of the review, and approved the draft protocol. AGS conducted initial and final searches. JU and JW screened titles, abstracts of publications identified by the searches, and screened whole paper of selected studies. RA, JU, and JW extracted data from selected studies. RA and JW assessed risk of bias in the included studies. All review authors interpreted the results and contributed to writing the paper with KW and JW leading. All authors contributed to the article and approved the submitted version.
