# Supplementary material for: Some home-based self-managed rehabilitation interventions can improve arm activity after stroke: A systematic review and narrative synthesis
Source: Front Neurol. 2023 Feb 2;14:1035256. doi: 10.3389/fneur.2023.1035256 (PMC9932529; doi:10.3389/fneur.2023.1035256)
Supplement: Supplementary file 1 [file Data_Sheet_1.docx]

**Appendix I: Search Strategies**

**PubMed (1809-present)**

(stroke[tiab] OR cva[tiab] OR cerebrovascular accident[tiab] OR cerebral infarction[tiab] OR brain infarction[tiab] OR brain ischemia[tiab] OR brain hemorrhage[tiab] OR brain embolism[tiab] OR brain thrombosis[tiab] OR cerebral embolism[tiab] OR cerebral thrombosis[tiab] OR stroke[mesh] OR “brain ischemia”[mesh]) AND ((telemedicine[tiab] OR telerehabilitation[tiab] OR home intervention[tiab] OR remote consultation[tiab] OR telehealth[tiab] OR telerehab[tiab] OR telemonitor*[tiab] OR teletherapy[tiab] OR remote monitoring[tiab] OR remote supervision[tiab] OR e-health[tiab] OR ehealth[tiab] OR e-therapy[tiab] OR etherapy[tiab] OR telesurveillance[tiab] OR mhealth[tiab] OR mobile health[tiab] OR self care[tiab] OR self management[tiab] OR telemedicine[mesh] OR “home care services”[mesh] OR “self care”[mesh]) OR ((home-based[tiab] OR web-based[tiab] OR self-guided[tiab] OR virtual[tiab] OR internet-based[tiab] OR videoconferenc*[tiab] OR web-supported[tiab] OR videoconferencing[mesh]) AND (rehab*[tiab] OR occupational therapy[tiab] OR physical therapy[tiab] OR “physical therapy modalities”[mesh] OR “stroke rehabilitation”[mesh] OR “exercise therapy”[mesh] OR “occupational therapy”[mesh]))) AND ((randomized controlled trial[pt] OR controlled clinical trial[pt] OR randomized[tiab] OR placebo[tiab] OR clinical trials as topic[mesh: noexp] OR randomly[tiab] OR trial[ti]) NOT (animals[mh] NOT humans[mh])) NOT ((child[mesh] OR infant[mesh] or adolescent[mesh]) NOT adult[mesh])

**Embase (embase.com, 1974-present)**

(stroke:ab,ti OR cva:ab,ti OR ‘cerebrovascular accident’:ab,ti OR ‘cerebral infarction’:ab,ti OR ‘brain infarction’:ab,ti OR ‘brain ischemia’:ab,ti OR ‘brain hemorrhage’:ab,ti OR ‘brain embolism’:ab,ti OR ‘brain thrombosis’:ab,ti OR ‘cerebral embolism’:ab,ti OR ‘cerebral thrombosis’:ab,ti OR ‘cerebrovascular accident’/exp OR ‘brain hemorrhage’/exp OR ‘brain infarction’/exp OR ‘brain ischemia’/exp) AND ((telemedicine:ab,ti OR telerehabilitation:ab,ti OR ‘home intervention’:ab,ti OR ‘remote consultation’:ab,ti OR telehealth:ab,ti OR telerehab:ab,ti OR telemonitor*:ab,ti OR teletherapy:ab,ti OR ‘remote monitoring’:ab,ti OR ‘remote supervision’:ab,ti OR ‘e-health’:ab,ti OR ehealth:ab,ti OR ‘e-therapy’:ab,ti OR etherapy:ab,ti OR telesurveillance:ab,ti OR mhealth:ab,ti OR ‘mobile health’:ab,ti OR ‘self care’:ab,ti OR ‘self management’:ab,ti OR telemedicine/exp OR telemonitoring/exp OR telerehabilitation/exp OR teletherapy/exp OR telehealth/exp OR ‘home care’/exp OR ‘home monitoring’/exp OR ‘home physiotherapy’/exp OR ‘home rehabilitation’/exp) OR ((‘home-based’:ab,ti OR ‘web-based’:ab,ti OR ‘self-guided’:ab,ti OR virtual:ab,ti OR ‘internet-based’:ab,ti OR videoconferenc*:ab,ti OR ‘web-supported’:ab,ti OR videoconferencing/exp) AND (rehab*:ab,ti OR ‘occupational therapy’:ab,ti OR ‘physical therapy’:ab,ti OR ‘stroke rehabilitation’/exp OR ‘occupational therapy’/exp OR ‘physiotherapy’/exp))) AND (‘randomized controlled trial’/exp OR ‘controlled clinical trial’/exp OR randomized:ab,ti OR placebo:ab,ti OR ‘clinical trial (topic)’/exp OR randomly:ab,ti OR trial:ti NOT ([animals]/lim NOT [humans]/lim)) NOT (([newborn]/lim OR [infant]/lim OR [child]/lim OR [adolescent]/lim) NOT ([adult]/lim OR [aged]/lim))

**Cochrane Central Register of Controlled Trials (Wiley)**

(stroke or cva or cerebrovascular accident or cerebral infarction or brain infarction or brain ischemia or brain hemorrhage or brain embolism or brain thrombosis or cerebral embolism or cerebral thrombosis) AND ((telemedicine or telerehabilitation or home intervention or remote consultation or telehealth or telerehab or telemonitor* or teletherapy or remote monitoring or remote supervision or e-health or ehealth or e-therapy or etherapy or telesurveillance or mhealth or mobile health or self care or self management) OR ((home-based or web-based or self-guided or virtual or internet-based or videoconferenc* or web-supported) AND (rehab* or occupational therapy or physical therapy)))

**CINAHL (Ebsco, 1937-present)**

(stroke or cva or cerebrovascular accident or cerebral infarction or brain infarction or brain ischemia or brain hemorrhage or brain embolism or brain thrombosis or cerebral embolism or cerebral thrombosis) AND ((telemedicine or telerehabilitation or home intervention or remote consultation or telehealth or telerehab or telemonitor* or teletherapy or remote monitoring or remote supervision or e-health or ehealth or e-therapy or etherapy or telesurveillance or mhealth or mobile health or self care or self management) OR ((home-based or web-based or self-guided or virtual or internet-based or videoconferenc* or web-supported) AND (rehab* or occupational therapy or physical therapy))) AND (TI double-blind OR AB double-blind OR TI random* assigned OR AB random* assigned OR TI control OR AB control OR TI placebo OR AB placebo)

**Physiotherapy Evidence Database (pedro.org.au)**

home-based stroke rehabilitation

stroke telerehabilitation

**OTseeker (otseeker.com)**

[Title/Abstract] like 'home-based rehabilitation' AND [Title/Abstract] like 'stroke'

[Title/Abstract] like 'stroke' AND [Title/Abstract] like 'telerehabilitation'

**REHABDATA (National Rehabilitation Information Center)**

home-based stroke rehabilitation

stroke telerehabilitation
